# Construction Notes

**Published:** 1905-09-09

**Authors:** 


### CONSTRUCTION NOTES.

A new out-patient's department has been opened at the Grimsby Hospital.

A proposition to establish a children's ward at the Pontypool Hospital is being considered.

The foundation-stone has been laid of a hospital in connection with the Royal Cornwall Sailors' Home at Falmouth. It will contain a main ward for six beds and two separate wards, convalescent-room, operating-room, and usual offices.

---

A new cottage hospital for Malton, Yorkshire, has been opened by the Countess Fitzwilliam. Earl Fitzwilliam provided a house for the purpose at a nominal rent and paid for the structural alterations necessary, and gave £100 to the hospital funds. There are two wards, with two beds in each, and a single ward, operating-theatre, offices, and matron's apartments.

A new Isolation Hospital has been opened at Penistone, in Yorkshire, for the use of the surrounding districts. The site was presented by Sir Walter Stanhope, and the institution will be known as the Stanhope Hospital. The cost of the building has been about £6,000. There is accommodation for eight scarlet fever and four typhoid cases. The administration block has been provided to serve for future extension.

---

The New Out-patient Hall was recently opened at the Grimsby and District Hospital. A memorial brass tablet was unveiled by Mr. T. W. G. Hewitt, son of the late William Taylor Hewitt, Esq., J.P., of old Weelsby Hall, by whose generosity the committee have been able to erect the new building. The late Mr. Hewitt bequeathed a sum of £1,250 to the hospital, which sum was devoted to building an out-patient department. A further expenditure of £1,600 was required, and this the trustees most generously consented to subscribe. The consequence is that the Grimsby and District Hospital now possesses a most handsome and up-to-date out-patient department. The new building consists of a large waiting-hall for out-patients, measuring 60 feet by 30 feet and 20 feet high and capable of seating 120 people, with casualty dressing-room, with lavatories, etc., surgeons' consulting-room, and rooms for men and women. The casualty and examining-rooms are fitted up with Doulton's special hospital sinks and are otherwise furnished with every requisite. The floors are of marble terrazzo, the walls having a high, glazed-brick dado and finished above in Portland and Parian cement, coloured with pale-green duresco. From the centre of the hall a short staircase leads to the hospital. The out-patients pass up this staircase to the dispensary and pass out through the exit-door in the corridor. Above the hall are built the nurses' new bedrooms, bath-room, and lavatory, on the lines suggested by The Hospital. Each nurse has a room to herself. The tablet which Mr. Hewitt unveiled bears the following inscription:—"This Out-patients' Department was erected with money bequeathed by William Taylor Hewitt, Esq., J.P., Weelsby Old Hall, who died on the 8th day of April, 1902." The Mayor unveiled a portrait of the Chairman, which had been subscribed for by the members of the Managing Committee. About 200 guests were present and the wards were tastefully decorated with plants and flowers by the nursing staff.

